# Risk factors for lymph node metastasis in patients with unifocal breast cancer

**DOI:** 10.1186/bcr2972

**Published:** 2011-11-04

**Authors:** A Robinson, M O'Keeffe, A Leaver

**Affiliations:** 1 Gateshead Health NHS Foundation Trust, Gateshead, UK; 2 Newcastle University, Newcastle, UK

## Introduction

In our Trust, in line with NICE guidance, all breast cancers undergo preoperative axillary ultrasound and, where indicated, needle testing. The current overall sensitivity of this process in our Trust is 57%. This study investigates tumour size, type and location as possible factors for patient triage to repeat preoperative axillary staging, aiming to increase our preoperative axillary staging sensitivity.

## Methods

This prospective study included all patients diagnosed and operated on for unifocal breast cancer in our Trust from September to December 2010. Descriptive statistics, chi-squared and logistic regression were performed upon data collated at MDT meetings.

## Results

Logistic regression of 101 females implies that as tumour size increases by 1 cm, nodal disease risk is 1.75 times larger, with 95% confidence limits. Comparing tumours <20 mm with those >20 mm, nodal disease risk is 5.818 times larger in the >20 mm group (*P *< 0.0005). No significant difference was found in nodal disease risk between the histological tumour types, although numbers of lobular and tubular carcinomas were small (*P *= 0.633). Data suggested a difference (0.27× smaller risk in UIQ versus UOQ) in probability of axillary node metastasis with tumour location with 95% confidence limits.

## Conclusion

This study demonstrates a clear and statistically significant association between tumour size and nodal disease. The data also suggest a difference in probability of nodal disease with different tumour location, although increased patient numbers are needed to confirm this. A larger trial for stratifying patients for single or double preoperative staging of the axilla is recommended.

